# Extending *in Silico* Protein Target Prediction Models to Include Functional Effects

**DOI:** 10.3389/fphar.2018.00613

**Published:** 2018-06-11

**Authors:** Lewis H. Mervin, Avid M. Afzal, Lars Brive, Ola Engkvist, Andreas Bender

**Affiliations:** ^1^Centre for Molecular Informatics, Department of Chemistry, University of Cambridge, Cambridge, United Kingdom; ^2^Cygnal Bioscience, Pixbo, Sweden; ^3^Hit Discovery, Discovery Sciences, IMED Biotech Unit, AstraZeneca, Gothenburg, Sweden

**Keywords:** target prediction, activation, inhibition, cheminformatics, functional effects, mechanism-of-action, chemical space, AD-AUC

## Abstract

*In silico* protein target deconvolution is frequently used for mechanism-of-action investigations; however existing protocols usually do not predict compound functional effects, such as activation or inhibition, upon binding to their protein counterparts. This study is hence concerned with including functional effects in target prediction. To this end, we assimilated a bioactivity training set for 332 targets, comprising 817,239 active data points with unknown functional effect (binding data) and 20,761,260 inactive compounds, along with 226,045 activating and 1,032,439 inhibiting data points from functional screens. Chemical space analysis of the data first showed some separation between compound sets (binding and inhibiting compounds were more similar to each other than both binding and activating or activating and inhibiting compounds), providing a rationale for implementing functional prediction models. We employed three different architectures to predict functional response, ranging from simplistic random forest models (‘Arch1’) to cascaded models which use separate binding and functional effect classification steps (‘Arch2’ and ‘Arch3’), differing in the way training sets were generated. Fivefold stratified cross-validation outlined cascading predictions provides superior precision and recall based on an internal test set. We next prospectively validated the architectures using a temporal set of 153,467 of in-house data points (after a 4-month interim from initial data extraction). Results outlined Arch3 performed with the highest target class averaged precision and recall scores of 71% and 53%, which we attribute to the use of inactive background sets. Distance-based applicability domain (AD) analysis outlined that Arch3 provides superior extrapolation into novel areas of chemical space, and thus based on the results presented here, propose as the most suitable architecture for the functional effect prediction of small molecules. We finally conclude including functional effects could provide vital insight in future studies, to annotate cases of unanticipated functional changeover, as outlined by our CHRM1 case study.

## Introduction

Target deconvolution is an important step in the subsequent analysis of data gleaned from phenotypic screenings, to identify the modulated targets of active compounds and enable the continued dissection of the biological processes involved in a system of interest (Terstappen et al., 2007; Raida, 2011; Kotz, 2012; Lee and Bogyo, 2013). One important additional parameter of consideration is the functional modulation of targets, since its activation or inhibition (in the simplest case of allowing only for two types of functional effects) may positively or negatively modulate a pathway, which in turn may relate in different ways to an observed phenotype (Parker et al., 1993; Bauer-Mehren et al., 2009; Dosa and Amin, 2016).

One example of this is Bone morphogenetic protein 1 (BMP1), which was identified as a key target linked to cytostaticity from a screening cascade discerning the cytotoxic and cytostatic tendencies of compounds (Mervin et al., 2016). In the absence of functional information for the respective target, and since activation of BMP signaling in prostate carcinoma cells is known to be cytostatic (hence its inactivation would not explain the observed phenotype) (Wahdan-Alaswad et al., 2012), the authors were forced to hypothesize that cytostatic agents *may* activate BMP1. Another study rationalized the polypharmacology of sleep-inducing compounds in rat, (which, without functional annotation) were forced to stipulate that bioactive compounds with multi-target activity *may* elicit their synergistic sleep parameter activity through inhibition of Histamine Receptor H1 (HRH1) and activation of Cholinergic Receptor Muscarinic 4 (CHRM4) (since the biological evidence at hand for both targets advocates this rationalization) (Drakakis et al., 2017). Sertindole, a withdrawn approved drug, was also experimentally determined within the study to changeover functional activity. Despite profiles linked to prolonged sleep bouts, the compound was linked to hyperactivity, not inhibition, at key targets implicated with increased bouts of sleep, which further demonstrates how the functional behavior of compounds needs to be considered to understand phenotypic response in biological systems.

One approach to target deconvolution is *in silico* target deconvolution, which is a well-established computational technique capable of inferring compound MOA by utilizing known bioactivity information (Koutsoukas et al., 2011; Wang et al., 2013; Lavecchia and Cerchia, 2016). This technique is well established in the areas for the deconvolution of phenotypic screens (Poroikov et al., 2001; Geronikaki et al., 2004; Liggi et al., 2014) and the identification of compound-side effects via bioactivity profiling of off-targets (Lounkine et al., 2012; Barton and Riley, 2016). The characterization of the functional effects of compounds is often a principle shortcoming for current *in silico* methods, since many protocols only provide probability for compound affinity at a target (Drakakis et al., 2013; Koutsoukas et al., 2013; Mervin et al., 2015).

Existing protocols, such as the Similarity Ensemble Approach (SEA) (Keiser et al., 2007) and Prediction of Activity Spectra for Substances (PASS) (Lagunin et al., 2000), provide functional annotation by training on a compound set extracted from the MDL Drug Data Report [MDDR] (2006). These implementations however only utilize active bioactivity data (experimentally validated negative bioactivity data are disregarded), which has been shown to hinder performance. Additional problems with MDDR are inconsistent annotation, since many activity classes are not on the target level (for example the activity class ‘anti-helminthic activity’) and relatively small numbers of compound-target pairs are available for modeling, compared to other current databases (Lagunin et al., 2000). Other cheminformatics approaches discriminate between agonist from antagonist classifications of ligands at nuclear receptors across targets simultaneously (within a single-model architecture) (Lagarde et al., 2017). This architecture could negatively affect performance due to the imbalance between the functional data and the requirement to assign probability scores across all target proteins.

We have in this work explored various cascaded approaches to predict the functional effects of orphan compounds and contrasted these with a single-model architecture (similar to previous approaches). To this end, we have assimilated a dataset of 22,836,983 compound-target annotations available in the Chemistry Connect (Muresan et al., 2011) repository across a range of G-protein-coupled receptors (GPCRs), Nuclear Hormone Receptors (NHRs), ion channels and transporters. The dataset comprises 817,239 binders (unknown if activating or inhibiting) and 20,761,260 non-binding compounds from binding assays, as well as 226,045 activating and 1,032,439 inhibiting compounds from functional assays, spanning a total of 332 protein targets.

**FIGURE 1:**
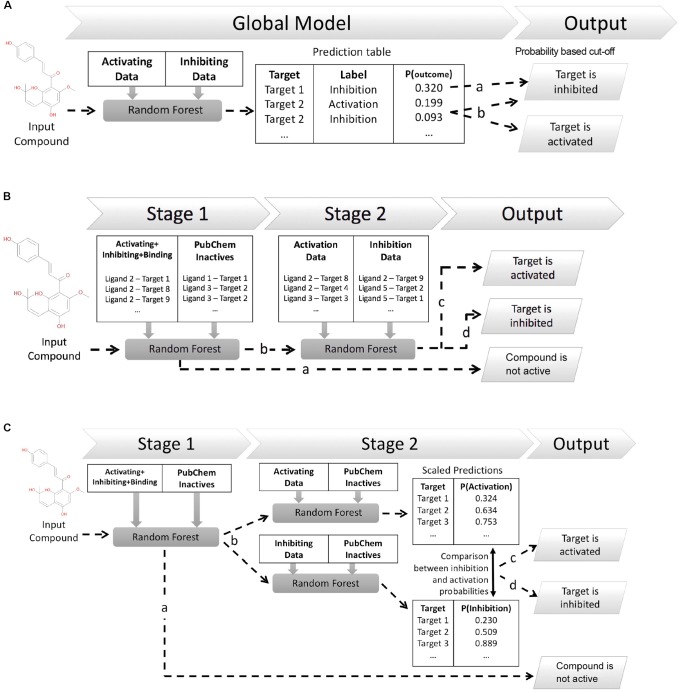
Different architectures employed for functional target prediction in this work. **(A)** Architecture 1 (Arch1). A single random forest (RF) algorithm is trained with the activating and inhibiting compounds across all targets. Model output is a list of functional predictions across all targets, ranked by probability for a target and corresponding functional label, ‘*p(target_outcome)*’. A probability threshold is employed to generate activating and inhibiting predictions. The functional label with the highest probability is selected if a compound is predicted as both activating and inhibiting, i.e., ‘Target 2’ (line *b*) is assigned the activating label if using a probability cut-off of 0.092. **(B)** Architecture 2 (Arch2). Stage 1 target prediction utilizes RF target prediction models trained using active (activating, inhibiting or binding only) and inactive compounds on a per target basis. Compounds predicted inactive during Stage 1 (line *a*) are removed from further cascading and annotated as inactive. Compounds predicted to be active during Stage 1 are subsequently profiled using Stage 2 functional prediction (line *b*), comprising RF models trained on the activating and inhibiting compounds on a per-target basis. Compounds are annotated as ‘*activating’* (line *c*) if the probability of activation is greater than inhibition (line *c*), or ‘*inhibiting’* if the probability of inhibition is greater than activation (line *d*). **(C)** Architecture 3 (Arch3). Stage 1 target prediction is employed in the same manner as Arch2. Compounds predicted to be active (line *b*) are subsequently profiled at Stage 2 using two independent RF models, trained using either activating or inhibiting compounds and an inactive compound set, and which are Platt scaled to ensure they are directly comparable. The probabilities of activation or inhibition generated by the two models and compared to deduce a functional prediction. A compound is annotated as ‘*activating’* if the likelihood of activation is greater than inhibition (line *c*), or as ‘*inhibiting’* if the probability of inhibition is greater than activation (line *d*). Although Arch2 and Arch3 enforce functional prediction in cases of both low activating and inhibiting probabilities, this is preferred for this study rather than the addition of an extra label (e.g., predicted binding only).

This work explores three different *in silico* architectures for functional target prediction which are summarized in **Figure 1**. **Figure 1A** outlines Architecture 1 (Arch1), a Random Forest (RF) algorithm trained with all functional labels across all targets within a single model [hence, an approach using only active (functional) data], which serves as a baseline to compare the cascaded, and hence more complex, architectures. Architecture 2 (Arch2), outlined in **Figure 1B**, is the first of two cascaded approaches, combining stage 1 target prediction with subsequent stage 2 functional prediction, which we rationalize could improve performance due to the cascaded nature of models. Stage 2 of Arch2 includes a single RF model trained on both activating and inhibiting compounds during stage 2. In comparison, **Figure 1C** depicts Architecture 3 (Arch3), which is based on an ensemble of two independent RFs trained on either activating or inhibiting compounds separately versus an inactive background set.

To establish the optimal model architecture, we conducted fivefold stratified cross validation for the three different modeling approaches. Models were also prospectively validated using an external testing set of 153,467 compounds, spanning 306 targets extracted from all functional in-house AstraZeneca data after a 4-month interim from initial training set extraction. The cross validation and time-split performance of the approaches has provided guidance into the choice of architecture to be deployed in-house for future triage processes.

## Materials and Methods

### Sources of Compound Training Data

AstraZeneca bioactivity data from Chemistry Connect (Muresan et al., 2011) was mined for functional data with bioactivities (IC_50_ and EC_50_) better than or equal to 10 μM and annotated with functional terms based on BioAssay Ontology (BAO) assay classifications (Vempati et al., 2012; Abeyruwan et al., 2014). The resulting dataset was filtered for the GPCR, NHR, ion channel and transporter targets, since they are considered to have the highest functional annotation accuracy (in-house) and encompass large numbers of activators which are not given in the case of enzymes.

The full complement of functional annotations includes various mechanisms, such as ‘activation,’ ‘antagonism,’ ‘inverse agonism,’ ‘opening,’ ‘closing’ and ‘modulation’ (full list shown in **Table 1**), which were chosen by BAO as the appropriate units to describe what each assay measures from assay endpoints. As a simple example, the unit EC_50_ was linked to ‘activation,’ whilst IC_50_ was annotated with ‘inhibition.’ More complex endpoints were assigned such that the measured activity of NHRs, GPCRs and ligand-gated ion channel mechanism-of-action (MOAs) were annotated as ‘agonist,’ ‘antagonist,’ or ‘partial antagonist,’ whilst voltage-gated ion channel MOAs were assigned ‘opening’ or ‘closing’ annotations.

**Table 1:** Functional mapping schema employed in this study.

Original BAO label	Simplified label
Activation	Activator
Agonism	Activator
Antagonism	Inhibitor
Blocking	Inhibitor
Closing	Inhibitor
Inhibition	Inhibitor
Inverse agonism	Inhibitor
Opening	Activator


*The functional labels of biological screens were reduced into the binary classifications of ‘activating’ or ‘inhibiting’ to reduce the complexity of the modeling in this study.*

In this study, we classified all compounds into the more simplified binary labels of ‘activating’ or ‘inhibiting’ endpoints using an internal mapping scheme (**Table 1**). Although imposing only two (activation and inhibition) functional labels may be an over-simplification, this is preferred to the complex situation resulting from the original complex BAO labeling, since it reduces training data into a binary problem per protein target, ensures larger numbers of compounds are retrained within each MOA, and that generated predictions are easily compared between the complete spectra of functional predictions between targets. It is also less algorithmically difficult to build classification models compared to regression, thereby usually improving performance.

**FIGURE 2:**
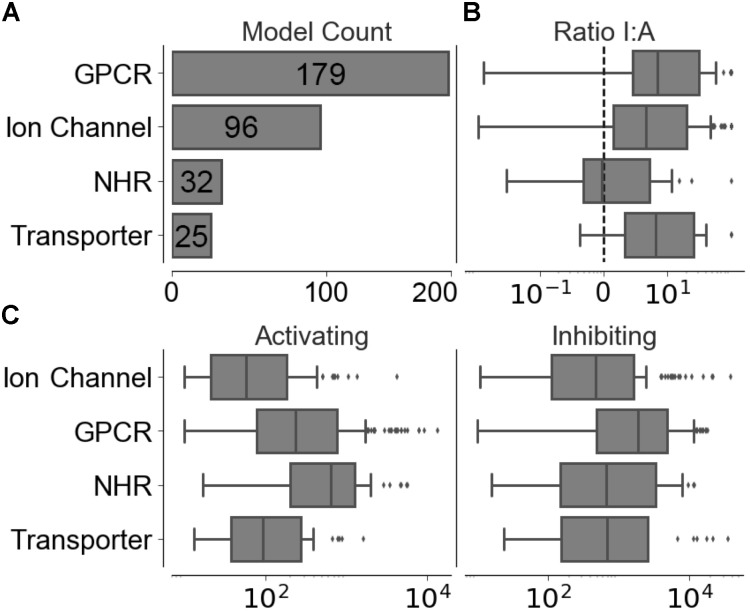
Distribution of training data across individual models in the four target protein classes modeled. **(A)** Number of targets modeled. **(B)** Ratio between inhibiting and activating compound-target data points. **(C)** Distribution of activating and inhibiting compounds available for training.

Compounds with conflicting activating and inhibiting annotations were removed from the training data. The resulting functional data set provided 226,045 activating and 1,032,439 inhibiting compounds spanning 320 different targets, the distribution of which is shown in **Figure 2A**, with a median of 186 ± 1,526 activating-target compound pairs and 1,190 ± 5,123 inhibiting-target compound pairs per target. The distribution of ratios between the functional labels (overall median ratio of 5.0 ± 27.4 inhibiting:activating compounds) and distribution of functional set sizes (overall median of 163 ± 1,462 and 948 ± 4,955 for the activating and inhibiting classes, respectively) are shown in **Figures 2B,C**.

Bioactivity data was also extracted from the same database, for compounds with binding activity (K_i_ or K_d_) better than or equal to 10 μM, as a supplementary source of training data for cascaded Stage 1 target prediction (Arch1 does not cascade predictions and so does not utilize binding information). The resulting data provided 817,239 binding compound-target pairs spanning 300 different targets, comprising a median and standard deviation of 752 ± 4,954 active compounds per target.

Non-binding (inactive) compounds were extracted from PubChem in a same manner as described in Mervin et al. (2015) which involved mapping to NCBI Gene IDs (GIDs) and Protein IDs (PIDs) to the Bioactivity Assay IDs (AIDs) held in the PubChem BioAssay repository for compounds annotated as ‘inactive’ in deposited bioactivity screens, using the PubChem REST (Kim et al., 2016) and PubChem PUG resources (NCBI, 2007). AstraZeneca high-throughput screens deposited in the HTS DataMart (an internal database of HTS information) were also mined for non-binding compounds from bioactivity screens with bioactivities (K_i_, K_d_, IC_50_, and EC_50_) greater than 10 μM. Compounds with conflicting non-binding annotations were removed from the training data.

To compile additional non-binding compounds for proteins not covered in the internal database or PubChem (hence, for cases where insufficient numbers of confirmed negatives were available), additional putative inactive compounds were sampled from PubChem using a sphere exclusion algorithm. In this protocol, compounds with a Tanimoto similarity coefficient (Tc) value of less than or equal to 0.4 are sampled as a background of putative inactive chemical space. Although sphere exclusion selection leads to artificially inflated performance, this is a necessary step to ensure the existence of a putative negative bioactivity class with sufficient coverage of inactive space to conduct target prediction. The resulting dataset includes 20,761,260 non-binders with a median of 32,320 ± 84,491 non-binding compound-target pairs per protein target.

Training compounds were subjected to pre-processing and filtered to retain targets with a minimum 10 activating and inhibiting compounds, to ensure only targets encompassing sufficient functional chemical space are retained for training. Although not essential for Stage 2 model training, binding data was also filtered for five or more compounds, to ensure the minimum number of binding data is equal to the number of folds used for cross validation. Supplementary Figure 1 shows a Venn diagram of the bioactivity data available for training, comprising 332 models. Overall, the training set includes 20,761,260 non-binding compounds, 817,239 binders, 226,045 activating and 1,032,439 inhibiting data points.

### Compound Pre-processing and Fingerprint Generation

RDKit (Landrum, 2006) (Version 2016.09.1) was employed to remove structures not containing carbon from the dataset, and to retain only compounds with atomic numbers between 21–32, 36–52, and greater than 53, as well as with a molecular weight between 100 and 1000 Da, to retain a more ‘drug-like’ (in the widest sense) chemical space. Compounds were standardized using an in-house (OEChem Toolkits, 2017) script, and RDKit was used to generate 2,048-bit (circular) Morgan fingerprints (Morgan, 1965), with the radius set to 2.

### *In Silico* Modeling

#### Single Model Functional Prediction (Arch1)

The first model architecture, Arch1 (shown in **Figure 1A**), utilizes a single RF trained using the activating and inhibiting data across all available targets, which is intended to serve as a baseline comparison against similar online web-based approaches such as SEA and PASS, which do not necessarily consider (non-)binding information or consider multiple functional labels within one model.

A RF classifier of 100 trees, with the number of features set to ‘*auto*’ and max depth set to ‘*20*,’ was implemented in Scikit-learn (Pedregosa et al., 2011), and trained using the binary matrix of activating and inhibiting compound fingerprints across all targets. The single-model provides a RF (class) probability (computed as the mean predicted class probabilities of the trees in the forest) of activating or inhibiting a target on an individual compound basis, when considering all other functional predictions for available targets. Generated probabilities are subsequently converted into binary predictions based on a probability cut-off [for example above 0.2 (line a) and 0.09 (line b) in **Figure 1A**], which is described in-depth throughout the next paragraph. The functional label with the highest probability is selected in situations when a target is considered both activating and inhibiting labels. For example, Target 2 would be considered activated when using a cut-off of 0.092 (as indicated by line ‘b’ in **Figure 1A**).

In order to compare Arch1 to the cascaded methods, a probability cut-off was applied to generate a final set of functional predictions from the probabilities generated. This threshold was defined as the probability providing the optimal F_1_-score performance (i.e., target or class performance averaged across the inactive, activating and inhibiting labels) from one percentile increments across the distribution of all scores obtained during cross validation and prospective validation, in a similar procedure to Perezgonzalez (2015). This is an important step since a robust method to fairly compare the different approaches is required, a topic which will be discussed in more detail in the section entitled “*Precision and recall versus. BEDROC and PR-AUC.*”

#### Stage 1 Target Prediction (Arch2 and Arch3)

Both Arch2 and Arch3 use Stage 1 target prediction. Here, input compounds are subjected to Stage 1 prediction and predicted as binding (or otherwise non-binding) based on the condition that the output probability of binding is greater than non-binding. Compounds predicted non-binding at this point are removed from the further cascaded profiling, whilst compounds predicted to bind are retained for Stage 2 functional prediction.

Stage 1 target prediction employs a similar target prediction protocol to the one described previously by Mervin et al. (2016) utilizing large scale inactive chemical space and active compounds from binding and functional assays. A RF classifier of 100 trees, with the number of features and max depth set to ‘*auto*’ and the ‘*class_weight*’ set to ‘*balanced*’ was implemented in Scikit-learn. The RF was trained using the binary matrix of inactive and active compound fingerprints on a per target bases, whilst supplying the ‘*sample_weight*’ parameter within the ‘*fit*’ method with the ratio of active and inactive training compounds. The implementation of stage 1 target prediction does not differ between Arch2 and Arch3.

##### Stage 2 prediction (Arch2)

Stage 2 prediction is employed in two different way between the different model architectures of Arch2 and Arch3. Both techniques aim to assign an activating or inhibiting functional prediction to input compounds predicted as active for a particular target during stage 1 prediction.

As visualized in **Figure 1B**, Arch2 employed two cascaded RF models overall (one RF for Stage 1 and one RF for Stage 2). The RF for Stage 2 used the same hyper-parameters as Stage 1, and was trained using the activating and inhibiting compound fingerprints on a per-target basis. This RF was calibrated using Platt Scaling using the Scikit-learn ‘*calibrate_classification_cv*’ method, with the number of calibration and validation folds set to ‘*3*’. Thus, the predictions generated by the Stage 2 RF can be directly interpreted as a likelihood that an input compound is an activator or inhibitor.

A functional prediction is made for the functional label with the largest probability output from the second cascaded model, i.e., if the probability of activation is higher than that for inhibition, then the compound is classified as an activator (and *vice-versa)*. Thus, this procedure does not distinguish for instances when no confident prediction can be made for the second cascaded prediction. This behavior is preferred for the purpose of this study, since enforcing a prediction for the highest label regardless of confidence ensures the output between Arch1, Arch2, and Arch3 can be compared within this study.

##### Stage 2 prediction (Arch3)

**Figure 1C** illustrates Arch3, which employed three RF models overall (one for Stage 1 and *two* independent RF models for Stage 2). Both Stage 2 RFs utilize the same parameters as in Stage 1, and are trained separately for activating and inhibiting compounds, respectively, versus a set of inactive compounds. Probabilities generated by both algorithms were calibrated using Platt Scaling via the Scikit-learn ‘*calibrate_classification_cv*’ method, with the number of calibration and validation folds set to ‘*3*’. Scaling the independent probabilities in this manner enables the comparison between the activating and inhibiting probabilities from both algorithms, even though the two are distinct models. Functional predictions are made for input compounds by selecting the activating or inhibiting label with the largest probability.

### Performance Measures: Precision and Recall versus BEDROC and PR-AUC

Although the Boltzmann-Enhanced Discrimination of the Receiver Operating Characteristic (BEDROC) (Truchon and Bayly, 2007) and Precision-Recall Area Under the Curve (PR-AUC) scores are frequently used to describe virtual screening performance, this is not an appropriate metric to compare the outputs between all the models benchmarked in this study. Such metrics are based on the distribution of probabilities for the classes for each method; however these are not comparable between the three architectures explored, since they are on different scales, represent different likelihoods, and are processed to generate an overall functional prediction in different ways.

For example, Arch1 is a *single model* with multiple labels hence the generated scores are low, since they are distributed over all 664 target-function effects which overall must sum to ‘1.0’. In comparison, Arch2 uses a binary classifier on a *per-target* basis for Stage 2, with hence only two probabilities are produced for activating or inhibiting, whose output sum to ‘1.0’. Thus, these values comprise comparatively higher values since they are shared between two output labels. Furthermore, Arch3 uses *two* different binary classifiers to deduce a final prediction in Stage 2, using the activating and inhibiting labels normalized with a background of inactive compounds. Thus, the probabilities of these activating and inhibiting labels do not sum to ‘1.0’, since they are distinct models. Therefore, we considered that precision, recall and F_1_-score (i.e., the actual output expected from the deployed models) are the most suitable and robust metrics to compare the performance of methods in the current situation.

### Cross Validation Methodology

Fivefold stratified cross validation was employed in Scikit-learn using the ‘*StratifiedKFold*’ method, ensuring training data is randomly shuffled and seeded. In this procedure, the non-binding and binding (only available for Arch2 and Arch3), and activating and inhibiting training data is split into five folds, whilst maintaining the ratio between compounds with different labels in each split. Each fold is used as a test and train set for cascaded Stage 1 and Stage 2 training and prediction. Binding data is only utilized within training sets for Stage 1 in the cascaded approaches, since it is only used to supplement Stage 1 training data and not employed during Stage 2.

The ranked list of functional compound predictions is used to calculate the optimal threshold for Arch1 (as discussed above) and used to generate precision, recall, and F_1_-score for Arch1, whilst the predicted outcome for the activating and inhibiting compounds from each test set is used to calculate the corresponding performance of the cascaded models. **Figure 2** gives details into the size of targets in terms of the data points available for modeling and ratio of inhibiting to activating compounds, which is known to influence the predictivity of target prediction models (Koutsoukas et al., 2013).

### Prospective Validation Data Set

AstraZeneca bioactivity data was mined in the same manner as described above after a 4-month interim (exactly the 4 months after extracting training data) to obtain an external dataset of compounds to prospectively validate the models. Compounds with affinities better or equal to 10 μM were extracted and employed for cascaded Stage 1 and Stage 2 prediction. The dataset includes a total of 63,640 activating and 89,827 inhibiting compounds for 306 targets (with the number of compounds per target classification shown in Supplementary Table 1), spanning both similar and dissimilar chemical space compared to the training set (prospective validation chemical space analysis shown in Supplementary Figure 2), with overall median Tc values of 0.51 ± 0.21 and 0.62 ± 0.19, respectively. Class-averaged precision, recall and F_1_-score were calculated for each architecture during temporal validation, since some targets comprise only very few test set compounds, which would hence produce unreliable performance metrics.

## Results

### Functional Data Available in AstraZeneca

We first analyzed the nearest-neighbor similarity distribution per-target for each classification, to explore the chemical space of the functional dataset and to rationalize to what extent the different sets of compounds can be distinguished in chemical similarity space and thus a rationale for implementing and evaluating functional target prediction models.

**FIGURE 3:**
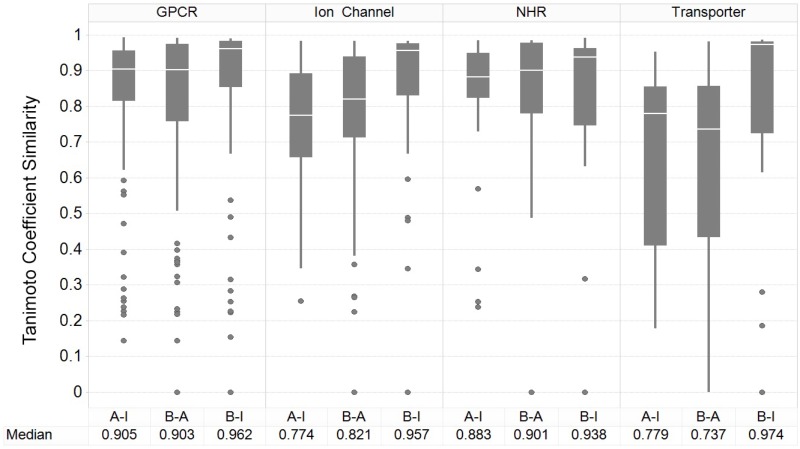
Nearest-neighbor Tanimoto similarity of active, activating and inhibiting compounds. ECFP_4 fingerprint similarity of the compounds to compare the three different bioactivity types, including comparison between activating versus inhibiting compounds (*A-I*), ligand binding-only (active) versus activating (*B-A*) and binding-only (active) versus inhibiting (*B-I*). The most similar compound was retained per-compound, per-target to indicate the nearest neighbor similarity. The overall distribution indicates that inhibiting compounds are more similar to their binding counterparts (*B-I*), in comparison to the other *A-B* and *B-A* comparisons.

**Figure 3** shows the results of the nearest-neighbor similarity distribution per-target for each classification. The overall distributions highlight that binding (active) and inhibiting compounds (“B-I”) are more similar to each other (median of 0.958) than both binding and activating (“B-A”) and activating and inhibiting (“A-I”) compounds (median similarities of 0.841 and 0.835, respectively). Overall, this analysis indicated there is some separation between the activating and inhibiting classes of compounds in chemical space, giving us a rationale for implementing and evaluating functional target prediction models (statistical analysis of chemical similarity between the target classes shown in Supplementary Table 2).

The GPCR class comprises the highest median NN similarity between the activating and inhibiting compounds of 0.905 (and an overall median of 0.923 between the three sets), a finding that is corroborated in literature since small structural modifications to GPCR-targeted ligands are known to convey major changes in their functional activity, converting agonists into antagonists and *vice-versa* (Dosa and Amin, 2016). Changes in certain moieties are shown to affect binding outcome more than others; for example, one study highlighted that steric modifications near a basic nitrogen, methylation of indoles, and aniline nitrogen substitutions appeared to play important roles in determining functional activity while keeping overall structure (as captured in the fingerprints employed in the current work) rather similar (Dosa and Amin, 2016). The close proximity between functional labels may be reflected in the performance of the models, since the overlap of features present in both sets confounds the separation between labels (Koutsoukas et al., 2013).

Nuclear hormone receptors are ranked as the second most similar target class based on the NN similarity between activating and inhibiting compounds, with a median Tc of 0.883. A range of ligand modifications can inter-convert functional activity due to changes in the directions in which these ligand R-groups are positioned within the ligand-binding domains (LBDs) of NHR cores (Huang et al., 2010). For example, one study explicitly outlined which ring system extensions alter the functional effects of activating compounds at the NHR estrogen receptor (ER), due to the protrusion of additional groups displacing the agonist conformation of α-helices in the LBD (Parker et al., 1993). In comparison, ion channels and transporters comprise comparatively dissimilar chemistry between compound sets, with median Tanimoto similarities of 0.774 and 0.779, respectively, between the activating and inhibiting compounds, giving rise to the expectation of better classification performance for those datasets.

### Cross Validation Results

We next performed stratified fivefold cross-validation (as described in the section “Materials and Methods”) and calculated precision, recall and F_1_-score metrics for 332 targets averaged over the fivefolds. Overall performance for each of the architectures was next calculated using the class-average precision and recall for the three functional labels (namely non-binding, inhibiting and activating) obtained over the 332 targets, the results of which are shown in **Table 2**.

**Table 2:** Target-averaged and class-averaged performance across the inactive, activating and inhibiting labels.

		Arch1 (optimal F_1_-score cut-off)		Arch2		Arch3	
				
		Precision	Recall	Precision	Recall	Precision	Recall
Cross validation	Target averaged	84.5 ± 12.1	68.7 ± 17.5	89.4 ± 9.8	79.2 ± 11.4	92.0 ± 9.1	82.9 ± 11.6
	Class averaged	76.1 ± 0.2	68.6 ± 0.9	89.3 ± 1.9	79.5 ± 2.7	91.9 ± 1.7	82.9 ± 3.4

Prospective validation	Class averaged	59.5 ± 3.2	48.1 ± 1.3	70.9 ± 4.0	52.9 ± 3.6	70.8 ± 3.5	53.1 ± 3.6
	Class averaged (Correct at Stage 1)	–	–	72.4 ± 3.3	71.0 ± 2.0	72.3 ± 2.8	71.3 ± 2.5


*Performance metrics are calculated by averaging the scores obtained over all targets or classes, for each of the three labels (inactive, activating, inhibiting), which are then averaged.*

It can be seen that the Arch1 architecture optimized for F_1_-score performed with overall class-averaged precision and recall performance of 84.5 ± 12.1 and 68.7 ± 17.5, respectively, which provides a baseline performance for what we expected to be superior (or certainly more complex) model architectures. This was indeed found to be the case, since Arch2 and Arch3 performed with target averaged precision and recall scores of 89.4 ± 9.8 and 79.2 ± 11.4, and 92.0 ± 9.1 and 82.9 ± 11.6, respectively (using a cut-off for the label with the largest probabilities as described in the section “Materials and Methods”).

In order to understand the performance distribution across different protein class labels, we next averaged precision, recall and F_1_-scores across the three functional labels for each of the GPCR, NHR, Ion Channel and NHR target classifications, as illustrated in the second row of **Table 2**. Overall, results from this analysis outlined that the baseline model performed with the lowest class averaged precision and recall scores of 76.1 ± 0.2 and 68.6 ± 0.9, whilst Arch2 performed with target class averaged precision and recall of 89.3 ± 1.9 and 79.5 ± 2.7, and Arch3 performed with the best scores of 91.9 ± 1.7 and 82.9 ± 3.4, respectively.

**FIGURE 4:**
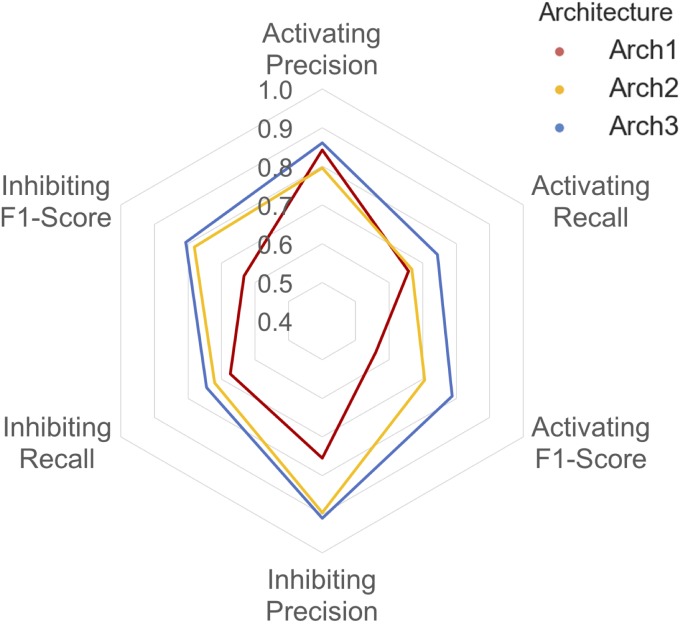
Inhibiting and activating class averaged performance of the three architectures during cross validation. The inactive, activating and inhibiting label performance for Arch1 (red), Arch2 (yellow) and Arch3 (blue) are shown. The performance profile of the three models illustrate that the two cascaded models outperform the single-model architecture. Arch3 is also shown to outperform Arch2, which is particularly evident for the activating recall and precision labels. Arch1 performance was calculated using the threshold comprising the highest overall F_1_-score.

A detailed breakdown of the protein target class averaged performance for the activating and inhibiting labels is shown in **Figure 4**. Overall, the inhibiting (more often majority) label performed with an overall class-averaged precision and recall of 75.5 and 67.3 for Arch1, 89.5 and 72.0 for Arch2 and 91.0 and 74.5 for Arch3. In comparison, the activating (more often minority) label performed with precision and recall scores of 84.2 and 65.8 for Arch1, 79.6 and 66.7 for Arch2, and 86.1 and 74.4 for Arch3, respectively. Hence, we conclude that Arch3 provides the optimal performance across the architectures assessed here.

Our results indicate models frequently perform with higher precision than recall (i.e., they are more certain about positive predictions they *do* make, than being able to identify compounds with the respective label across all of chemical space). Although Arch2 and Arch3 provide overall superior performance profiles, Arch1 exhibits superior activating precision (84.2) over Arch2 (79.6). We attribute this to the fact that Arch1 relies solely on activating or inhibiting compounds, and hence a more simplistic input space compared to Arch 2 and Arch3, which results in a larger number of incorrectly predicted activating compounds with fewer positive predictions with a greater propensity to be correct.

Arch2 and Arch3 also exhibit lower recall compared to precision, which is a consequence of the two-stage functional prediction, when false-negative binding predictions from Stage 1 are not used as input for Stage 2 prediction. Our findings also indicate that Arch3 can best handle the imbalance between inhibiting and activating labels compared to Arch2, to obtain higher activating recall and precision performance, a trend which will be discussed in more detail in the following.

In order to test if the activating and inhibiting performance of Arch3 models lie above that of the Arch2 approach (and hence there is statistical value in normalizing the models using a background of inactive compounds when cascading predictions), we next conducted a two-sample Kolmogorov–Smirnov (KS) test for the precision, recall and F_1_-score values obtained for Arch2 and Arch3 (overall results are summarized in the following, more detailed results are shown in Supplementary Table 3). The KS test produces p-values less than 0.05 (5% confidence threshold) for the activating precision, recall and F_1_-score (3.96^E-04^, 7.90^E-05^, and 1.95^E-05^, respectively) and inhibiting F_1_-score (4.93^E-03^), indicating that Arch3 performance is statistically improved for these performance parameters, compared to the Arch2 model architecture.

**FIGURE 5:**
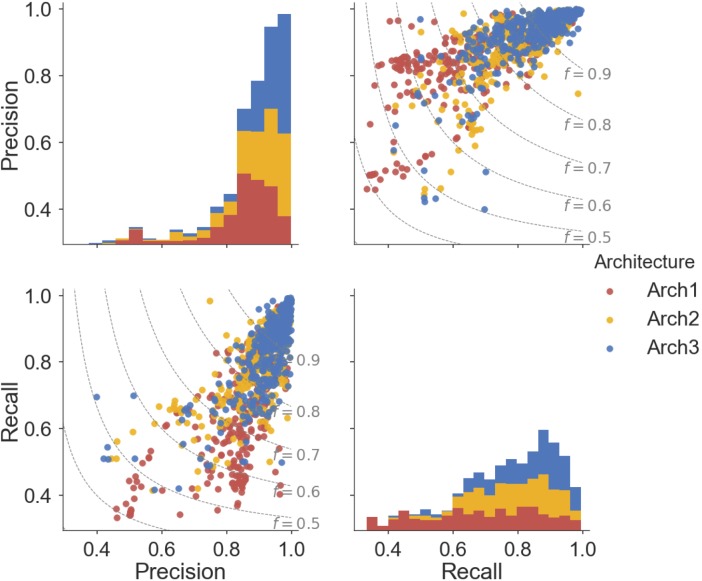
Pairwise distribution of the relationship between precision and recall scores for each architecture. Subplot grids in the upper right and lower left cells visualize a scatter plot of the relationship between recall and precision along with F_1_-score boundaries (*f*). Diagonal plots in upper left and lower right show stacked histograms of the precision or recall scores achieved by the Arch1 (red), Arch2 (yellow), and Arch3 (blue) architecture. Our results show that Arch3 provides the highest performance, for models with both high precision and recall, with a higher distribution of scores above the F_1_-score 0.9 boundary line.

Overall, ∼50% (166) of the Arch2 and ∼64% (214) of the Arch3 models performed with precision and recall values greater than or equal to 0.8, as shown in **Figure 5**. Thus, functional effects of compounds can be predicted with respectable performance for over half the target modeled. Conversely, only ∼40% (133) of the Arch1 models performed with equivalent precision and recall values above 0.8, as shown by the lower distribution of scores.

In total, eight targets failed to predict activating or inhibiting molecules using Arch2 and hence received precision and recall values of ‘0’ (shown as outliers in **Figure 5**). Seven of the eight targets were assigned such scores since no predictions were generated for the activating label, with five of these targets comprising fewer than 25 activating training instances and an average of 92.7 inhibiting compounds for every activating compound (92.7:1 ratio). In comparison, there was an equivalent ratio of 15.6:1 for the models that *worked*, with F_1_-score above 0.8. Hence, we conclude here that the poor performance in these situations was due to the domination of the inhibition class and lack of sufficient data points for the minority (activating) class, and conclude that datasets comprising 25 compounds constitute the minimum to generate bioactivity models with the architectures employed here.

We next analyzed how the Arch3 architecture handles class imbalance with superior class averaged precision, recall and F_1_-score performance, which is shown in Supplementary Figure 3. It can be seen that this architecture performs with superior performance than Arch2 and Arch1, with all models comprising one or more inhibiting predictions, and only one model with relatively few activators (18) failing to predict any activating molecules. Since this observation is likely a result of the independent comparison of activating or inhibiting compounds with an inactive background set and the subsequent comparison of Platt scaled probabilities, our most likely explanation is that this, combined with the Platt scaling, enables the minority (more often activating) class to assign higher confidence to predictions to surpass the majority (more often inhibiting) functional label predictions.

We next sought to identify the performance of the activating and inhibiting labels for the Arch2 and Arch3 architectures separated by the individual target classifications, as shown in Supplementary Figure 4. Our results demonstrate that the distribution of performance differs between classes, where the high performance of the GPCRs and NHRs (averaged median F_1_-scores of 86.8 and 84.3, respectively) can be contrasted with transporters, and comparatively poorly performing ion channels (with averaged median F_1_-scores of 77.5). Although the poor performance for ion channels and transporters may be unexpected due to the overall rather high separation in chemical space between activating and inhibiting training compounds (**Figure 3**), the large imbalance between the labels (as previously outlined by the median activating versus inhibiting ratios of 6.95 and 6.58, highlighted in **Figure 2B**) is likely one reason for the poor performance of these classes, particularly when considering activating label performance.

In order to identify further factors influencing performance of the predictivity of models, we next explored the impact of training set size of data points with functional annotations, the similarity of the five nearest intra-target neighbors and overall cross-validation F_1_-score performance as depicted in Supplementary Figure 5. The figure demonstrates both increasing nearest-neighbor similarity within activating and inhibiting compounds and overall model size are shown to improve model performance, with a large proportion of data points clustered toward the top right hand corner of the 3D plot. The intra-target similarity of the models is shown to increase in accordance with training set size, with increased likelihood to cover similar compounds in the train and test set (which hence leads to increased performance). In comparison, small models (with fewer than 100 compounds) perform with more diverse performance (standard deviation of 18), due to the decreased chance of retaining similar compounds throughout the cross validation.

The models also exhibit higher variance in nearest neighbor similarity due to the reduced coverage of chemical space (as previously shown in Supplementary Figures). Smaller target models below 100 compounds with similar nearest neighbors (Tanimoto similarity above 0.6) are shown to perform better, supporting the view that targets with few activating or inhibiting compounds can be reliably utilized in functional target prediction models, providing similar chemistry to the compounds which predictions are made for is represented within the training set. These findings are at least partly due to the nature of cross-validation, and the fact that data is comprised from a single source and that in larger classes there is greater chance to have analogs (which are then easier to predict).

This analysis (Supplementary Figure 5) also highlights the influence of the modeling approach on the cross validated performance of the models, with blue and red markers denoting the Arch2 and Arch3 approaches, respectively. 97 (∼30%) of the cascaded models have an F_1_-score greater than 0.95, with 63 (∼65%) of these originating from the Arch3 approach, illustrating the superior performance of this method compared to the Arch2 method. The figure illustrates both Arch2 and Arch3 approaches perform erratically in situations with low intra-target similarity and small size.

### Prospective Validation

The performance of the functional prediction protocols was next analyzed using an external data set extracted from functional screens available at AstraZeneca after a 4-month intermission from the initial date of training data mining. The overall class averaged precision and recall results for the non-binding, binding and inhibiting labels achieved during prospective validation are shown in **Table 2**. Arch1 performed with a class-averaged precision and recall of 59.5 ± 3.2 and 48.1 ± 1.3. In agreement with cross-validation results, the cascaded models performed with superior precision and recall, where Arch2 achieved a precision and recall of 70.9 ± 4.0 and 52.9 ± 3.6, whilst Arch3 performed with values of 70.8 ± 3.5 and 53.1 ± 3.6, respectively. Therefore, a cascaded model architecture produces more predictive models both during cross validation, as well as when applied to a prospective data set comprising novel areas of chemical space (Supplementary Figure 2).

**FIGURE 6:**
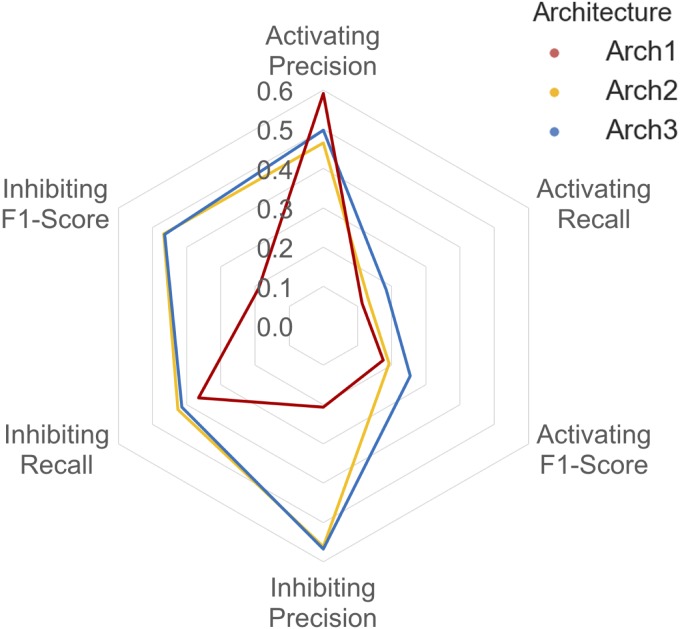
Inhibiting and activating class averaged performance during prospective validation. Arch1 (red) generates a distinct performance profile separate from the cascaded architectures, where Arch2 (yellow) and Arch3 (blue) exhibit significantly reduced class averaged inhibiting precision (and hence markedly lower F_1_-score these labels). This is likely due the inability of the single model architecture to counter for the imbalance between the majority (inhibiting) and minority (activating) labels, since Arch1 is forced to consider all functional labels across all targets at once. This factor ranks inhibiting labels higher within the ranked list of predictions, producing higher numbers of false positive inhibiting predictions, and thus reduces the precision of the inhibiting label for this architecture.

The class-averaged precision, recall and F_1_-score performance split between functional labels for prospective validation is shown in **Figure 6**. Our findings show that although the Arch1 architecture outperforms Arch2 and Arch3 based on activating precision (by a margin of ∼0.90 and ∼0.12 respectively), the cascaded models far outperform the inhibiting precision score obtained by Arch1, by a margin of ∼0.35 for both architectures. The inhibiting and activating recall are also higher for the Arch2 and Arch3 models, and hence produce higher F_1_-scores for both cascaded architectures compared to Arch1, with scores of ∼0.19 and ∼0.26 for the activating label and ∼0.47 and ∼0.46 for the inhibiting compounds, respectively. These findings are likely due to the single model architecture of Arch1, since the single-model architecture creates many false inhibiting predictions due many large classes with inhibiting data, which hence dominate the model with higher probabilities.

In comparison to cross validation, the difference in Arch2 and Arch3 precision, recall and F_1_-score performance is narrowed for prospective validation. For example, cross validation results showed a margin of ∼0.40 and ∼0.51 between activating and inhibiting target class averaged precision and recall values, which are reduced to ∼0.19 and ∼0.20 during external validation testing.

The cascaded models have fewer compounds for Stage 1, with hence less chemical space, and hence more false negatives. This is shown *via* the striking distribution of poor Arch2 and Arch3 recall, particularly for the activating compounds, where 87 targets (∼59% of these belong to the GPCR class) failed to predict true-positive active compounds (i.e., ‘predicted to bind’) during Stage 1 target prediction. The removal of testing instances are consequently assigned recall scores of ‘0’. This problem is further exacerbated by the imbalance of the external testing set between functional compounds, as indicated by the ratio between prospective validation compounds, which is applied to already imbalanced models.

Given this observation, we next assessed only *the fraction of active compounds predicted to be positives at Stage 1* for Arch2 and Arch3 (according to the protocol outlined in **Figure 1**), to give a better indication for the benchmarked performance between the two different cascaded methods of Stage 2 prediction (i.e., only compounds predicted active at line *b* in **Figures 1B,C** were considered for this part of the analysis). As shown in **Table 2**, this analysis produces class averaged recall scores for Arch2 and Arch3 of 72.4 ± 3.3 and 71.0 ± 2.0 versus 72.3 ± 2.8 and 71.3 ± 2.5, respectively, indicating the recall and F_1_-score performance of is higher for Arch3 than Arch2 when benchmarking cascaded Stage 2 performance by considering only true positives from Stage 1 predictions.

To further explore in more detail the performance of different target classifications between Arch2 and Arch3, we analyzed the distribution of F_1_-score prospective validation performance when only active compounds predicted to be positives at Stage 1 are considered. Supplementary Figure 7 also shows, in a similar trend to cross validation, that the ion channels and transporter class have a distribution of activating scores lower than the GPCR and NHR classes due to the imbalance between the activating and inhibiting compounds also represented in the external testing set, whilst there is higher performance for the inhibiting classification of compounds due to the domination of this label.

**FIGURE 7:**
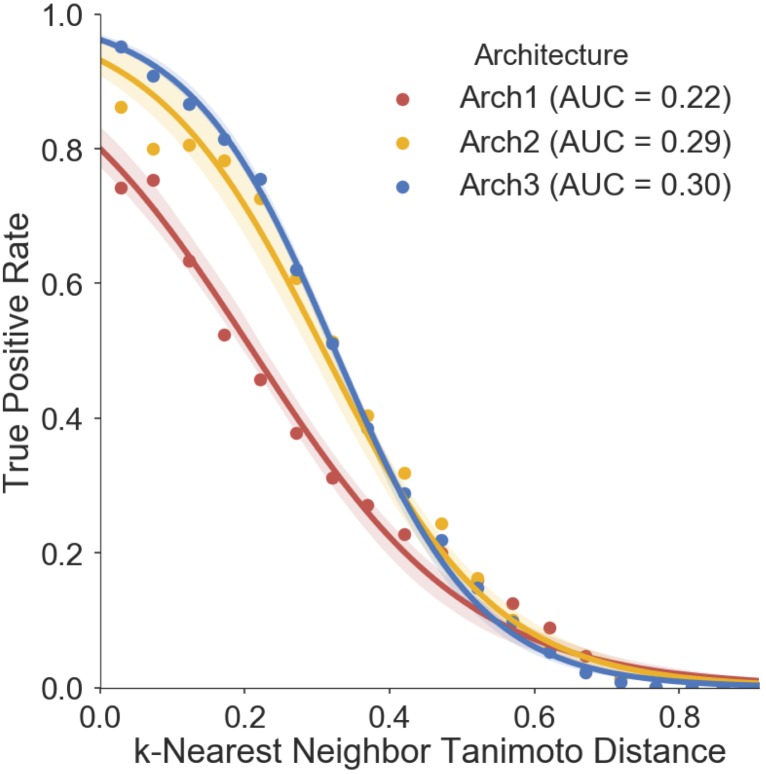
Prospective validation distance-based applicability domain (AD) analysis. AD curves are shown for Arch1 (red), along with Arch2 (yellow) and Arch3 (red). Each line performs with AUC scores of 0.22, 0.29, 0.30, respectively, indicating that Arch3 performs with overall superior AUC when considering the true positive rate achieved and increasing distance between training and prospective validation compounds. The Arch1 architecture produces similar true positive rates to the cascaded architectures for distances beyond 0.6, indicating that all three model architectures have difficulty in extrapolating into novel areas of the chemical space. True positive rate is defined as the recall of the activating and inhibiting data points for each distance bin.

We finally assessed the applicability domain (AD) of all model architectures using ‘distance to the training set’ as a method (Gadaleta et al., 2016; Hanser et al., 2016), the results of which are shown in **Figure 7**. The averaged five nearest neighbors (*k* = 5) in the training set and the true positive rate (TPR) (defined by the frequency of correct predictions within activating and inhibiting testing compounds) are shown for Arch1, Arch2 and Arch3. We see that the TPR decreases in accordance with increasing dissimilarity from the nearest compound in the respective label of training data across all architectures, as expected, with Arch3 performing with the highest area under the applicability domain curve (AD-AUC) of 0.30. This analysis enables us to assign confidence to novel predictions as follows; for example, an input compound with a near neighbor similarity between 0.8 and 0.85 would have an anticipated true-positive rate of ∼35% for Arch1, ∼78% for Arch2 and ∼81% for Arch3. We can also see that although Arch1 performs with a comparatively low AD-AUC of 0.22, all architectures obtain comparatively similar TPR rates throughout increasing dissimilarity scores from 0.6 onward, and hence models are unable to extrapolate into these dissimilar areas of chemical space.

In a final case study, we analyzed the aforementioned study of Drakakis et al. (2017), to illustrate a scenario where functional prediction would have added value to a computational study. In this work, target prediction profiles were related to prolonged sleep bouts, where changing functional effects on receptors was related to the change on the sleep effect of compounds. Contrary to the reasoning gained from the *in silico* mechanism-of-action analysis, Sertindole, which was expected to increase sleep bouts, actually increased wakefulness by 44.9 min. In the absence of functional prediction, the authors hypothesized that the compound switched functional activity at one of the key receptors (CHRM1), compared to the other sleep inducing compounds (Alcaftadine, Ecopipam, Cyproheptadine, and Clopenthixol), leading to hyperactivity and promoted wakefulness. We hence suggest that our method could improve similar analyses by providing vital insight into cases of unanticipated functional changeover.

To illustrate this, we profiled the functional activity of Sertindole at the CHRM1 receptor using Arch1, Arch2, and Arch3. Arch2 and Arch3 predictions both indicate target specific *activation* of CHRM1, compared to the four sleep inducing compounds above. Arch1 however, did not predict CHRM1 activation or inhibition, and thus would not have predicted any functional activity against the CHRM1 receptor.

We conclude that this case study highlights how cascaded functional models provide vital insight into this previous work, and that the unanticipated functional activity could have helped to direct resources toward the experimental functional testing of CHRM1, which was not conducted in the original study.

## Discussion

In this study, we present an in-depth analysis of functional bioactivity data available in-house. We first analyzed the chemical space of functional data, to rationalize whether the functional sets of compounds can be distinguished using chemical similarity. Binding and inhibiting compounds were more similar to each other [median Tanimoto Similarity (Tc) of 0.958] than both binding and activating or activating and inhibiting compounds (median Tc of 0.841 and 0.835, respectively). There was separation between functional sets giving us a rationale for implementing and evaluating functional prediction models. We first generated Architecture 1 (Arch1), which uses a simplistic RF similar to existing approaches, and contrasted this with two forms of cascaded models, namely Arch2; comprising a Stage 2 model trained directly on the activating and inhibiting compounds, and Arch3; comprising two independent Stage 2 models trained on either activating or inhibiting compounds, and a set of inactive compounds, respectively. Fivefold cross validation and temporal validation was performed using data available at AstraZeneca after a 4-month interim. Cross validation highlighted Arch3 achieved the highest precision, recall and F_1_-scores, which we attributed to the independent comparison of activating or inhibiting compounds with the inactive background sets, and the subsequent comparison of Platt scaled probabilities. In comparison, Arch1 had the lowest precision and recall performance which we attributed to the single-model architecture. Prospective validation indicated that Arch2 and Arch3 outperform the Arch1 overall and hence outlined there is benefit in cascading predictions using a more complex model architecture. Distance-based applicability domain (AD) analysis outlined Arch3 achieved superior AD-AUC (area under the AD curve) and hence superior extrapolation into novel areas of chemical space. Models will be deployed in-house to aid with future phenotypic screening analyses. We conclude that predicting functional effects could provide vital insight for future studies, to annotate cases of unanticipated functional changeover, as outlined by our CHRM1 case study.

## Author Contributions

LM assimilated the functional data sets, implemented and evaluated the algorithms presented in this work, and wrote this manuscript. AMA helped to implement design model architectures and benchmarking. LB curated the BAO functional dataset. AB and OE conceived the main theme on which the work was performed and made sure that scientific aspect of the study was rationally valid. All authors contributed to revising the final draft of the manuscript.

## Conflict of Interest Statement

LB was employed by company AstraZeneca and currently works at Cygnal Bioscience. OE is employed by company AstraZeneca. The remaining authors declare that the research was conducted in the absence of any commercial or financial relationships that could be construed as a potential conflict of interest.
