# Supplementary material for: Extending in Silico Protein Target Prediction Models to Include Functional Effects
Source: Front Pharmacol. 2018 Jun 11;9:613. doi: 10.3389/fphar.2018.00613 (PMC6004408; doi:10.3389/fphar.2018.00613)
Supplement: Supplementary file 1 [file Data_Sheet_1.docx]

Supplementary Material

Extending in silico protein target prediction models to include functional effects

Lewis H. Mervin^1^, Avid Azfal^1^, Lars Brive^2^, Ola Engkvist^3^ and Andreas Bender^1*^

*** Correspondence:** Andreas Bender: ab454@cam.ac.uk

# Supplementary Figures


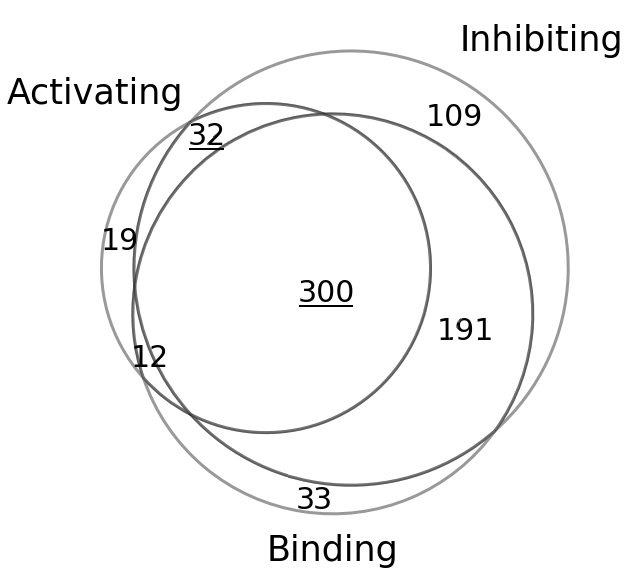


**Supplementary Figure 1. The overlap of models between the activating and inhibiting classification of bioactive compounds.** These numbers are generated after employing a threshold of at least ten bioactivities for the activating and inhibiting compounds and a minimum five compounds for active annotations across all GPCRs, NHRs, ion channels and transporters (although actives from binding assays are only used to supplement functional activities, the number of binding compounds must equal the number of folds during cross validation if they are to be used).There are *300* targets with all three annotations, whilst *32* targets comprise only activating and inhibiting functional annotation categories (both underlined). Taken together, this overlap produces a dataset of *332* targets with adequate amounts of training data for the protocol presented in this study.

**
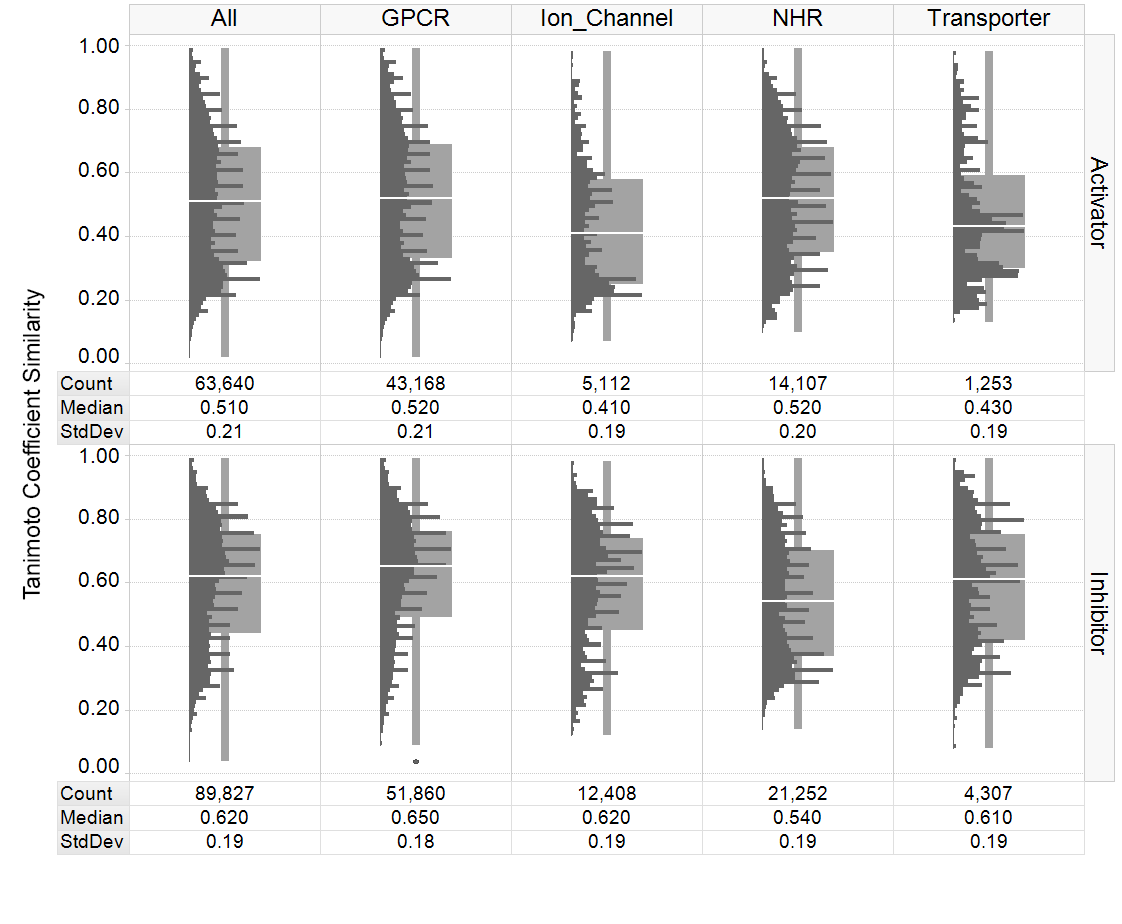
**

**Supplementary Figure 2. Similarity between training and prospective testing data.** Testing compounds span both similar and dissimilar realms of chemical space compared to training sets, enabling the gradual and robust benchmarking of the applicability domain (AD). Overall, activating and inhibiting compounds have a median Tc test-train similarity of 0.51 and 0.62, which highlights the challenging nature of the prospective validation set.


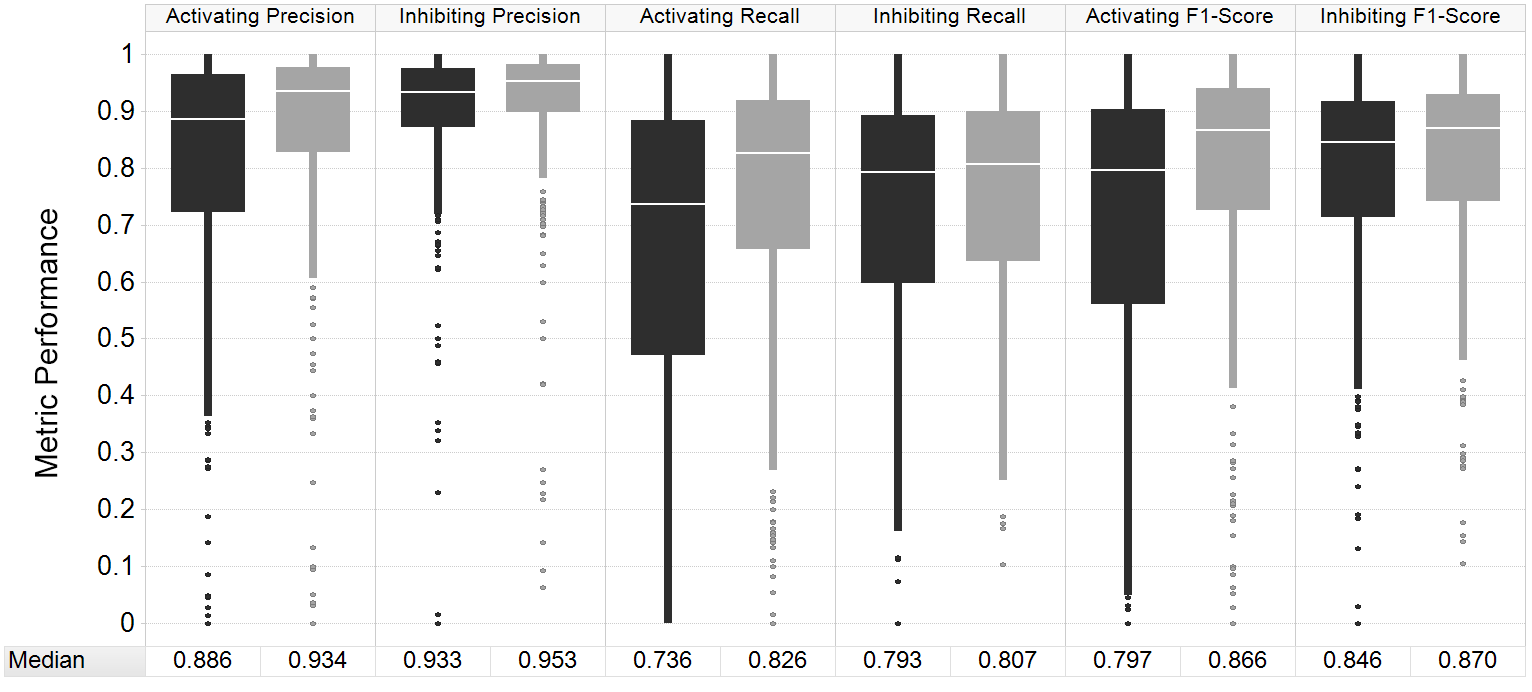


**Supplementary Figure 3. Cross validated performance of the Arch2 and Arch3 approaches**. Distribution of target performance indicates the Arch3 approach (light grey) outperforms the Arch2 (dark grey) during 5-fold cross validation when considering the distribution of precision, recall and F_1_-scores.

**
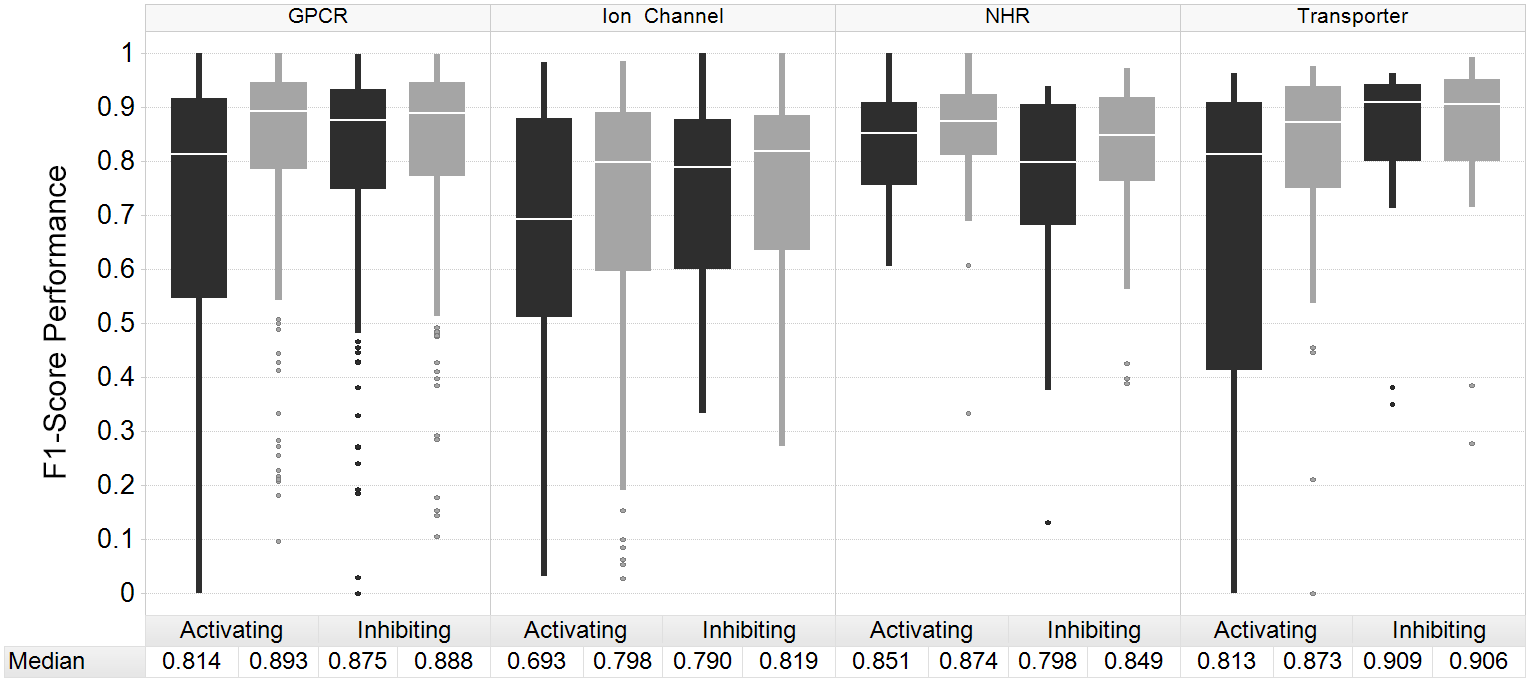
**

**Supplementary Figure 4. Arch2 and Arch3 activating and inhibiting performance across different protein families.** GPCR and NHR targets comprise a higher distribution of F_1_-Scores compared to ion channels and transporters, which may be a result of the imbalance of training data between the activating and inhibiting compounds for these targets. The Arch3 architecture (light grey) particularly performs with superior performance compared to Arch2 (dark grey) in these cases, indicating that the Arch3 can better correct the for class imbalance between the two labels.


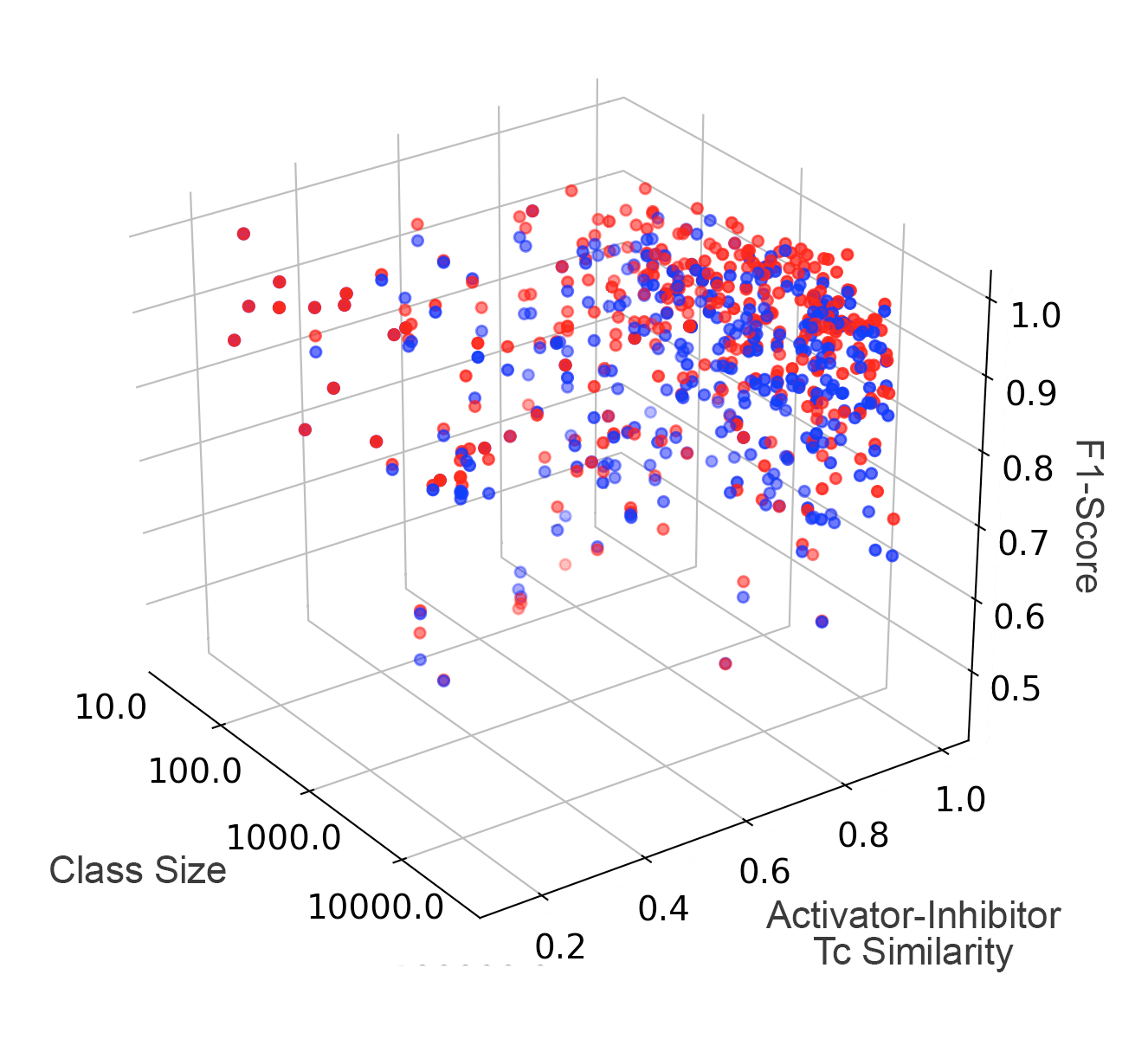


**Supplementary Figure 5. Influence of model size, model similarity on Arch2 and Arch3 cross validation F_1_-Score performance.** Arch2 and Arch3 architectures are shown in blue and red, respectively, with transparency set to increase with distance from the view. Models with greater numbers of training compounds correlate with increased nearest neighbor similarity and higher F_1_-score performance. Larger numbers of red markers are placed towards the top of the plot, illustrating the superior performance of the Arch3 architecture when considering a balance between recall and precision.


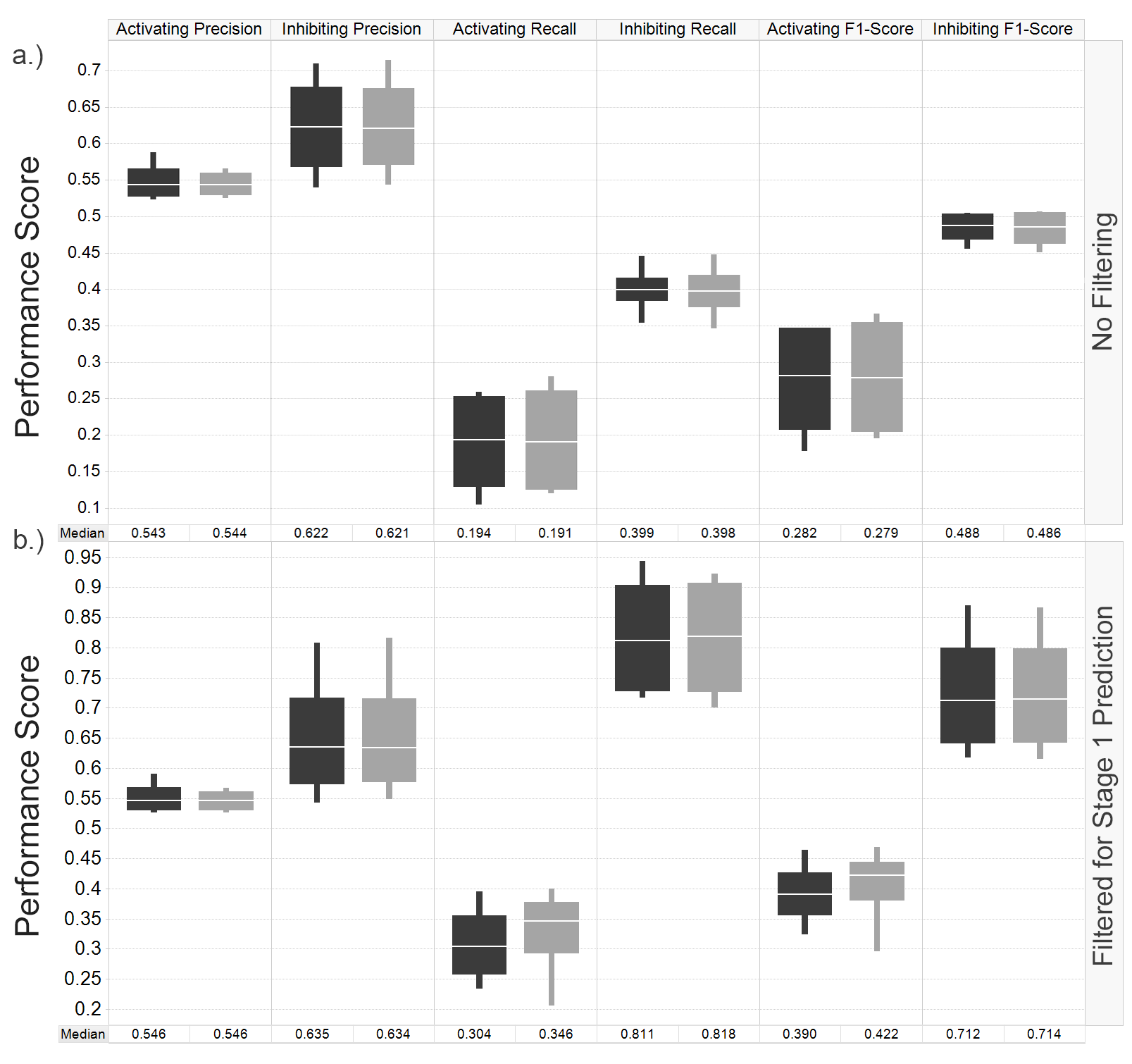


**Supplementary Figure 6. a.) Prospective validation of the Arch2 and Arch3 approaches**. Distribution of performance metrics indicate Arch2 (dark grey) and Arch3 (light grey) perform with similar prospective validation performance. Thus, the gap between the two approaches are narrowed when compared to cross validation, which is due to the increased Stage 1 false negative rate caused by dissimilar testing compounds which decreases the resolution for the gleaned performance for the Arch2 and Arch3 methods. **b.)Prospective validation filtered for Stage 1 predicted active compounds**. Filtering for Stage 1 true positive target predictions increases the measured performance among the different metrics, enabling us to better benchmark the differences between the two architectures. Upon filtering, Arch3 now performs with improved activating recall and F_1_-Score compared to Arch2. Overall, Arch2 and Arch3 perform with precision and recall values of 60.3% and 56.5% and 60.2% and 57.0%, respectively


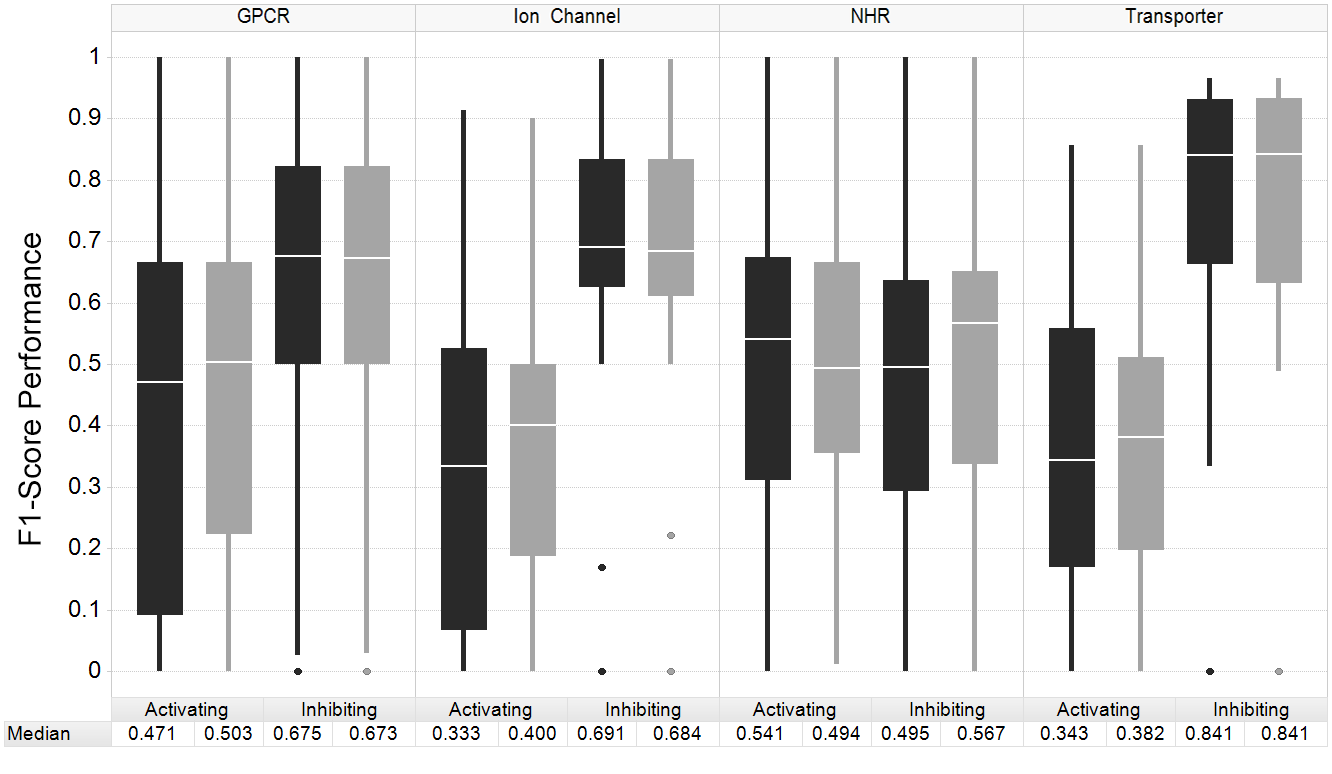


**Supplementary Figure 7. Performance of Stage 2 (functional effect) validation, based only on compounds predicted to bind to the respective target in Stage 1.** Prospective validation of compounds with true positive predictions at Stage 1 target prediction are shown between the different target classes. Ion channels and transporters perform with the lowest activating F_1_-Score in both the Arch2 (dark grey) and Arch3 (light grey) architectures, similar to the cross validation. This is caused by the class imbalance between the activating and inhibiting compounds in both the training and prospective.

# SupplementaryTables

## Supplementary Table 1. In-house data available for prospective validation. Bioactivity data available since the initial extraction of functional data was employed as a prospective validation set, with the complete number of data points available for testing shown in the “Total No. Prospective Validation Comps” column. These compounds were subjected to cascaded functional prediction. Activating and inhibiting compounds predicted to be inactive at Stage 1 can be filtered from the analysis, to give the numbers in the “Filtered No. Prospective Validation Comps” column.

|  | **Total No. Prospective**  **Validation Comps** | | **Filtered No. Prospective**  **Validation Comps** | | |
| --- | --- | --- | --- | --- | --- |
|  | **Activating** | **Inhibiting** | **Activating** | **Inhibiting** | **Inhibiting:Activating Ratio** |
| GPCR | 43,168 | 51,860 | 27,430 | 31,608 | 1.15 |
| NHR | 14,107 | 21,252 | 10,703 | 11,668 | 1.09 |
| Ion Channel | 5,112 | 12,408 | 2,989 | 5,646 | 1.89 |
| Transporter | 1,253 | 4,307 | 494 | 1,615 | 3.27 |
| **TOTAL** | **153,467** | | **92,153** | |  |

## Supplementary Table 2. a). Compare distributions in each target class by Kruskal-Wallis test to test if the distributions are similar to each other. Results show that the distribution of compound similarities for GPCRs and Ion Channels reject the null hypothesis (indicated with *via* ‘*’ character), i.e. that the distribution of similarities between the categories of compound activities (inhibiting, activating or binding-only) are statistically different.b). Pairwise comparison of the similarity distributions between the significant Ion Channels and GPCR target classes using the Kolmogorov-Smirnov test. For both Ion Channels and GPCRs, the compound similarities between activating versus inhibiting compounds are significantly different from the other sets (indicated with *via* ‘*’ character).

a.)

| **Target Classification** | **Rejects null hypothesis** | **Corrected p-values** |
| --- | --- | --- |
| GPCR | True | 8.95E-05* |
| Ion Channel | True | 3.95E-09* |
| NHR | False | 8.21E-01 |
| Transporter | False | 1.10E-01 |

b.)

| **Target Class** | **Set 1** | **Set 2** | **Ks two sample Result** | **P-value** |
| --- | --- | --- | --- | --- |
| GPCRs | Activating vs Inhibiting | Binding versus activating | 1.28E-01 | 9.58E-02 |
|  | Activating vs Inhibiting | Binding versus Inhibiting | 3.07E-01 | 5.70E-08* |
|  | Binding Versus Activating | Binding versus Inhibiting | 2.29E-01 | 1.28E-04* |
| Ion Channels | Activating vs Inhibiting | Binding versus activating | 1.35E-01 | 3.19E-01 |
|  | Activating vs Inhibiting | Binding versus Inhibiting | 4.58E-01 | 1.57E-09* |
|  | Binding Versus Activating | Binding versus Inhibiting | 4.38E-01 | 1.01E-08* |

## Supplementary Table 3. Kolmogorov-Smirnov test for the improvement of Arch2 and Arch3 scores obtained from cross-validation. Results from the two-sample Kolmogorov-Smirnov (KS) test indicate the precision, recall and F_1_-score of activating labels and the precision of the inhibiting label (shown in bold) are statistically increased when using a Arch3 over the Arch2. The two-sampled test was conducted using ‘mstats.ks_2samp’ in Scipy, with the alternative hypothesis specified as ‘*greater*’. Significant *p-values* (less than 5.0E-02) enable the rejection of the null hypothesis, that the Arch3 model performances are not greater from Arch2 scores.

|  |  | Arch2 vs. Arch3 Comparison | | | |
| --- | --- | --- | --- | --- | --- |
|  |  | Activating | | Inhibiting | |
|  |  | KS statistic | P-value | KS statistic | P-value |
| Metric | Precision | 0.154 | **3.96E-04** | 0.127 | **4.93E-03** |
|  | Recall | 0.169 | **7.90E-05** | 0.048 | 4.63E-01 |
|  | F_1_-score | 0.181 | **1.95E-05** | 0.072 | 1.76E-01 |
